# A numerical scheme for the ground state of rotating spin-1 Bose–Einstein condensates

**DOI:** 10.1038/s41598-021-02249-4

**Published:** 2021-11-23

**Authors:** Sirilak Sriburadet, Yin-Tzer Shih, B.-W. Jeng, C.-H. Hsueh, C.-S. Chien

**Affiliations:** 1 Department of Applied Mathematics, National Chung Hsing University, Taichung, 402 Taiwan; 2 Department of Mathematics Education, National Taichung University of Education, Taichung, 403 Taiwan; 3 Department of Physics, National Taiwan Normal University, Taipei, 11677 Taiwan

**Keywords:** Applied mathematics, Mathematics and computing

## Abstract

We study the existence of nontrivial solution branches of three-coupled Gross–Pitaevskii equations (CGPEs), which are used as the mathematical model for rotating spin-1 Bose–Einstein condensates (BEC). The Lyapunov–Schmidt reduction is exploited to test the branching of nontrivial solution curves from the trivial one in some neighborhoods of bifurcation points. A multilevel continuation method is proposed for computing the ground state solution of rotating spin-1 BEC. By properly choosing the constraint conditions associated with the components of the parameter variable, the proposed algorithm can effectively compute the ground states of spin-1 $^{87}Rb$ and $^{23}Na$ under rapid rotation. Extensive numerical results demonstrate the efficiency of the proposed algorithm. In particular, the affect of the magnetization on the CGPEs is investigated.

## Introduction

Experimental results on rotating spinor Bose–Einstein condensates (BEC)^1–3^ have intrigued researchers on theoretical physics and applied mathematics to study quantum phenomena of superfluidity, such as hexagonal vortex lattice and square vortex lattice, which do not exist in a single component BEC. Comprehensive investigation of spinor and rotating BEC can be found in the review articles^4,5^ and the book^6^, respectively. Recent investigation on this topic can be found, e.g., in^7–11^. More precisely, Nolan et al.^7^ presented a model of a spin-squeezed rotation sensor utilizing the Sagnac effect in a spin-1 BEC in a ring trap. Gautam and Adhikari^9^ exploited variational method and numerical solution to study the vortex-bright solitons in a quasi-two-dimensional spin-orbit-coupled hyperfine spin-1 BEC using the mean-field theory. Eto et al.^10^ studied the dynamics of BEC of $^{87}Rb$ atoms with hyperfine spins of 1 and 2. Based on the mean-field theory, the dynamics of rotating spinor BEC at ultra-cold temperatures can be described by the nonlinear Schrödinger equations (NLS), or the coupled Gross–Pitaevskii equations^12–14^ (CGPEs) as follows:

1 $$\begin{aligned} \displaystyle {\mathbf{i}}\hbar \partial _t\varphi _1({\mathbf{x}}, t)&=  {} \displaystyle \left[ -\frac{\hbar ^2}{2m}\nabla ^2+V({\mathbf{x}}) +E_1+c_0|\Phi |^2+ c_2(|\varphi _1|^2+|\varphi _0|^2-|\varphi _{-1}|^2) -\omega \widehat{L}_z\right] \varphi _1 \nonumber \\&\quad + c_2{\varphi }_{-1}^*\varphi _0^2,\nonumber \\ \displaystyle {\mathbf{i}}\hbar \partial _t\varphi _0({\mathbf{x}}, t)&=  {} \displaystyle \left[ -\frac{\hbar ^2}{2m}\nabla ^2+V({\mathbf{x}}) +E_0+c_0|\Phi |^2+c_2(|\varphi _1|^2+|\varphi _{-1}|^2)-\omega \widehat{L}_z\right] \varphi _0 \nonumber \\&\quad +2c_2\varphi _{-1}{\varphi }_0^*\varphi _1,\nonumber \\ \displaystyle {\mathbf{i}}\hbar \partial _t\varphi _{-1}({\mathbf{x}}, t)&=  {} \displaystyle \left[ -\frac{\hbar ^2}{2m}\nabla ^2+V({\mathbf{x}}) +E_{-1}+c_0|\Phi |^2+c_2(|\varphi _{-1}|^2+|\varphi _0|^2-|\varphi _1|^2) -\omega \widehat{L}_z\right] \varphi _{-1} \nonumber \\&\quad + c_2\varphi _0^2{\varphi }_1^*. \end{aligned}$$

Here $\Phi ({\mathbf{x}}, t)$ $= (\varphi _1({\mathbf{x}}, t)$, $\varphi _0({\mathbf{x}}, t)$, $\varphi _{-1}({\mathbf{x}}, t))^T$ denotes the three-component wave function, $|\Phi |^2=|\varphi _1|^2+|\varphi _0|^2+| \varphi _{-1}|^2$, ${\mathbf{x}}=(x,y,z)$ the state/space variable, *t* the time variable, $\hbar$ the Planck constant, *m* the atomic mass, $V({\mathbf{x}})=\frac{m}{2}(\omega _1^2x^2 + \omega _2^2y^2 +\omega _3^2z^2 )$ the external trapping potential with $\omega _1$, $\omega _2$ and $\omega _3$ being the trapping frequencies in the *x*-, *y*- and *z*-direction, respectively, and $E_j$ $(j = -1, 0, 1)$ the Zeeman energy of hyperfine spin component $m_F = l$ $(l = -1, 0, 1)$ in the uniform magnetic field. The constants $c_0=\frac{4\pi \hbar ^2}{3m} (a_0+2a_2)$ and $c_2=\frac{4\pi \hbar ^2}{3m}(a_2-a_0)$ denote the spin-independent and spin-exchange interactions, respectively, where $a_j$ is the *s*-wave scattering lengths for the channel of total hyperfine spin *j* $(j=0,2)$, $\omega$ is an angular velocity, and $\widehat{L}_z=\hbar \cdot L_z$, where $L_z= -{\mathbf{i}}(x\partial y-y\partial x)$ is the *z*-component of the angular momentum. Moreover, the superscript “$*$” over the components $\varphi _i$ of the wave functions denotes the complex conjugate. Notice that the interaction is either repulsive or attractive depending on the constant $c_0$ is positive or negative. Furthermore, the spin-exchange interaction can be antiferromagnetic or ferromagnetic depending on the constant $c_2$ is positive or negative. The GPE based on the mean-field theory is treated as the model for describing the physical phenomena of BEC at zero temperature. However, there are still several models which have been proposed to take into account finite temperatures effects in a quantum fluid. A well-known example is the Zaremba–Nikuni–Griffin (ZNG) method^15,16^, in which a dissipative GPE for the condensate wave function is coupled with a heat base described by the Boltzmann equation. Another simpler model is the so-called stochastic projected Gross–Pitaevskii equation (SPGPE), in which thermal fluctuations of the bosonic field are taken into account at stochastic forcing^17–19^.

For completeness we define the state of lowest energy of a BEC system with fixed number of particles as the ground state. The states with energies greater than the ground-state energy are called excited states. The linear Zeeman (LZ) energy and the quadratic Zeeman (QZ) energy are given by^20–22^.

2 $$\begin{aligned} p_0 = -\frac{1}{2}(E_1-E_{-1}), \end{aligned}$$

and

3 $$\begin{aligned} q_0 = -\frac{1}{2}(E_1+E_{-1}-2E_0), \end{aligned}$$

respectively. Both the parameters $p_0$ and $q_0$ play important roles in the ground state phase diagram as well as the dynamics of spin-1 condensates. From Eqs. (2) and (3) we have

4 $$\begin{aligned} E_1 = -p_0-q_0+E_0,\quad E_{-1} = p_0-q_0+E_0. \end{aligned}$$

Various numerical methods have been proposed for computing the ground state solution of both one- and two-component rotating BEC^23–33^. In particular, the preconditioned imaginary time evolution method (PITEM), or the so-called continuous normalized gradient flow (CNGF) was widely used. See^34,35^. Recently, the performance of PITEM and continuation methods on some test problems in boson-fermion mixtures was compared in^36^. Published articles on numerical study of spin-1 BEC is also abundant. See e.g.,^37–40^. Research papers on numerical investigation of rotating spin-1 BEC can be found e.g., in^18,19,41–47^.

We consider rotating spin-1 BEC with a strong confinement in the *z*-direction, i.e., $\omega _2\approx \omega _1$ and $\omega _3\gg \omega _1$. Assume that the separation of variables is available for the wave function $\Phi$. That is, $\Phi (x,y,z,t)=\varPhi (x,y,t)\phi _{g}(z)$, where $\varPhi (x,y,t)$ denotes the 2D ground state wave function, and $\phi _{g}(z)$ is a 1D ground state wave function which is a harmonic oscillator in the *z*-direction. We integrate the energy functional over *z*. Then from Eq. (4) the 3D CGPEs (1) can be reduced to the following quasi-2D CGPEs^48^:

5 $$\begin{aligned} \displaystyle {\mathbf{i}}\partial _t\varphi _1&=  {} \displaystyle \left[ -\frac{1}{2}\nabla ^2+V({\mathbf{x}})-p-q +g_n\widetilde{N}|\varPhi |^2+g_s\widetilde{N}(|\varphi _1|^2 +|\varphi _0|^2-|\varphi _{-1}|^2)-\omega L_z\right] \varphi _1\nonumber \\&\quad + g_s\widetilde{N}{\varphi }_{-1}^*\varphi _0^2,\nonumber \\ \displaystyle {\mathbf{i}}\partial _t\varphi _0&=  {} \displaystyle \left[ -\frac{1}{2}\nabla ^2+V({\mathbf{x}})+g_n\widetilde{N}|\varPhi |^2 +g_s\widetilde{N}(|\varphi _1|^2+|\varphi _{-1}|^2)-\omega L_z\right] \varphi _0+ 2g_s\widetilde{N}\varphi _{-1}{\varphi }_0^*\varphi _1,\nonumber \\ \displaystyle {\mathbf{i}}\partial _t\varphi _{-1}&=  {} \displaystyle \left[ -\frac{1}{2}\nabla ^2+V({\mathbf{x}})+p-q+g_n\widetilde{N}| \varPhi |^2+g_s\widetilde{N}(|\varphi _{-1}|^2+|\varphi _0|^2 -|\varphi _1|^2)-\omega L_z\right] \varphi _{-1}\nonumber \\&\quad + g_s\widetilde{N}\varphi _0^2{\varphi }_1^*, \end{aligned}$$

where ${\mathbf{x}}=(x,y)$, $g_{n}=\frac{4\pi }{l_{3}\sqrt{2\pi }}(a_{0}+2a_{2})$, $g_{s}=\frac{4\pi }{l_{3}\sqrt{2\pi }}(a_{2}-a_{0})$ with $l_{3}=\sqrt{\frac{\hbar }{m\omega _{3}}}$, $V({\mathbf{x}}) =\frac{1}{2}(x^2+y^2)$, and the LZ and QZ terms are scaled according to $p=p_0/\hbar \omega _3$, $q=q_0/\hbar \omega _3$, and $\widetilde{N}$ is the total number of particles in the condensates. Indeed, the effect of dimension reduction emerges as the ratio of $\omega _1/\omega _3\approx \omega _2/\omega _3$. However, the validity of dimension reduction is based on the extremely strong confinement along the reduced direction, say, the *z*-direction. The strength of confinement is inversely proportional to the trapping frequency $\omega _3$, and consequently $\omega _3$ does not vanish in the quasi-2D scenario. Two key features of Eq. (5) are the normalization or mass of the wave function

6 $$\begin{aligned} \widetilde{N}(\varPhi (\cdot ,t)):=\Vert \varPhi (\cdot ,t)\Vert ^2 :=\int _{{\mathbb {R}}^2}\sum ^1_{l=-1}|\varphi _l({\mathbf{x}},t)|^2 d{\mathbf{x}}\equiv \widetilde{N}(\varPhi (\cdot ,0))=1,\ t>0, \end{aligned}$$

as well as the magnetization

7 $$\begin{aligned} M(\varPhi (\cdot ,t)):=\int _{{\mathbb {R}}^2}\left[ |\varphi _1({\mathbf{x}},t)|^2 -|\varphi _{-1}({\mathbf{x}},t)|^2\right] d{\mathbf{x}}\equiv M(\varPhi (\cdot ,0))=M, \ |M|\le 1. \end{aligned}$$

Denote the energy per particle by $E(\varPhi (\cdot ,t))\equiv E(\varPhi (\cdot ,0)),t>0$. We consider the stationary state wave function $\Psi ({\mathbf{x}})$ $=(\psi _1({\mathbf{x}}),$ $\psi _0({\mathbf{x}}),$ $\psi _{-1}({\mathbf{x}}))^T$ and define the Lagrangian by

8 $$\begin{aligned} {\mathcal {L}}(\Psi , \mu , \lambda ) := E(\Psi )-\mu (\Vert \psi _1\Vert ^2 +\Vert \psi _0\Vert ^2+\Vert \psi _{-1}\Vert ^2-1)-\lambda (\Vert \psi _1\Vert ^2-\Vert \psi _{-1}\Vert ^2-M), \end{aligned}$$

where the parameters $\mu$ and $\lambda$ in Eq. (8) are the Lagrange multipliers with respect to the chemical potential and magnetic potential of rotating spin-1 BEC, respectively. The ground state solution of rotating spin-1 BEC is obtained by minimizing the energy functional $E(\Psi )$ subjected to the constraints Eqs. (6) and (7). Note that the LZ energy term *p* can be absorbed into the magnetic potential because of conservation of the total magnetism in the system^49–51^. Recently, the effect of quadratic Zeeman energy was considered both experimentally^12^ and theoretically^13,14^ in a spin-1 BEC, where new vortices were observed. Substituting the formulae

9 $$\begin{aligned} \varphi _l({\mathbf{x}},t)=e^{-i\mu _l t}\psi _l({\mathbf{x}}), \ l=1,0,-1, \end{aligned}$$

into Eq. (5), we obtain the following Euler–Lagrange equation as follows:

10 $$\begin{aligned} \mu _{1}\psi _1&=  {} \left[ -\frac{\displaystyle 1}{\displaystyle 2}\nabla ^2 +V({\mathbf{x}})-q+g_n\widetilde{N}|\Psi |^2-\omega L_z\right] \psi _1\nonumber \\&\quad +g_s\widetilde{N} \left[ (|\psi _1|^2+|\psi _0|^2-|\psi _{-1}|^2)\psi _1 + {\psi }_{-1}^*\psi _0^2\right] ,\nonumber \\ \mu _0\psi _0&=  {} \left[ -\frac{\displaystyle 1}{\displaystyle 2}\nabla ^2 +V({\mathbf{x}})+g_n\widetilde{N}|\Psi |^2-\omega L_z\right] \psi _0\nonumber \\&\quad +g_s\widetilde{N} \left[ (|\psi _1|^2+|\psi _{-1}|^2)\psi _0 +2\psi _{-1}{\psi }_0^*\psi _1\right] ,\nonumber \\ \mu _{-1}\psi _{-1}&=  {} \left[ -\frac{\displaystyle 1}{\displaystyle 2} \nabla ^2+V({\mathbf{x}})-q+g_n\widetilde{N}|\Psi |^2-\omega L_z\right] \psi _{-1}\nonumber \\&\quad +g_s\widetilde{N} \left[ (|\psi _0|^2+|\psi _{-1}|^2-|\psi _{1}|^2)\psi _{-1} + {\psi }_1^*\psi _0^2\right] , \end{aligned}$$

where $$\mu _1=\mu + \lambda ,\ \mu _0=\mu ,\ \mu _{-1}=\mu - \lambda $$, and $$|\Psi |^2=|\psi _1|^2+|\psi _0|^2+|\psi _{-1}|^2$$.

To compute the ground state solution of BEC using PITEM^34–36^ or CNGF^27,30,37,38^, the parameters $\mu , \lambda$ and $\widetilde{N}$ are fixed. However, in numerical continuation methods these parameters are treated as variables, which will change gradually as the continuation proceeds. See, e.g.,^36^ and further references cited therein. The Lyapunov–Schmidt reduction^52^, Chap. 7, is a popular technique to deal with nonlinear eigenvalue problems in bifurcation theory, and has been widely used to study the existence of the ground state and excited states of NLS. See e.g.,^53–59^. In particular, Charalampidis et al.^57^ proposed a deflated continuation algorithm which can discover novel solution branches of the nonlinear system. Chang et al.^58^ applied the Lyapunov–Schmidt reduction combined with continuation methods to study numerical solutions of NLS. Xu et al.^59^ exploited the Lyapunov–Schmidt reduction to study the existence of solitary waves of two-component BEC. The branching of nontrivial solution curves from eigenvalues of the associated linear eigenvalue problem was discussed in^60^.

In this paper, we investigate the existence of nontrivial solution curves of the CGPEs using the Lyapunov–Schmidt reduction. By performing a small perturbation of the cubic nonlinearity, we show how the nontrivial solution curves branching from bifurcation points on the trivial one. Our result is a 2D and three-component generalization of that in^59^. Next, we describe a novel multi-level continuation algorithm to compute the ground states of Eq. (10) for various values of the parameters, where the Fourier sine functions are used as the basis functions to discretize the CGPEs. In the first two levels of the algorithm we use the chemical potential $\mu$, and then add the magnetic potential $\lambda$ as the first and the second continuation parameters, respectively. In our numerical computations we consider the cases with magnetization $M=0$ and $M\ne 0$. For convenience we omit the quadratic Zeeman energy *q* in Eq. (10). Note that the numerical computations of the ground states for spin-1 BEC with quadratic Zeeman energy has been widely investigated in^40^. Instead of using $M = 0$ as the constraint condition in Eq. (7), we impose a more reflexible one

11 $$\begin{aligned} \int _{{\mathbb {R}}^2}\left[ |\psi _1({\mathbf{x}})|^2-|\psi _{-1}({\mathbf{x}})|^2\right] d{\mathbf{x}} -M\Vert \Psi \Vert ^2 = 0 \end{aligned}$$

in the second level, where $M\in [0,1]$ is fixed, and the $L_2$-norm $\Vert \cdot \Vert$ is defined by

12 $$\begin{aligned} \Vert \Psi \Vert ^2:=\int _{{\mathbb {R}}^2}\left[ \,|\psi _{-1}({\mathbf{x}})|^2 +|\psi _0({\mathbf{x}})|^2+|\psi _1({\mathbf{x}})|^2\,\right] d{\mathbf{x}} =\Vert \psi _{-1}\Vert ^2+\Vert \psi _{0}\Vert ^2+\Vert \psi _{1}\Vert ^2. \end{aligned}$$

Note that as we start to switch from the trivial solution curve to the nontrivial one of Eq. (10), the two-norm of the components $\psi _1$ and $\psi _{-1}$ are relatively small compared to the value $M \ne 0$. It is impossible that Eq. (7) will hold. Thus we multiply the magnetization *M* in Eq. (11) by $\Vert \Psi \Vert ^2$ in the continuation process in order to keep Newton’s method from divergence in the corrector step of the continuation algorithm. In the third level of the algorithm we intend to use the number of particles $\widetilde{N}$ as the third component of the parameter variable. Since the scales of $\mu , \lambda$ and the number of the particles $\widetilde{N}$ are quite different, we impose an artificial parameter $\nu$ as the third component of the parameter variable to control the increment of $\widetilde{N}$. We will also apply the proposed algorithm to study how the wave function of Eq. (10) changes with respect to the angular velocity when $\omega >1$, where we impose a harmonic plus quartic trap on the system. Note that the numerical computations for the ground states of fast rotating spin-1 BEC become difficult when the angular velocity $\omega >1$, and it is getting more challenging as $\omega$ increases. To our knowledge, the physical phenomena of the ground states of rotating spin-1 BEC with $M>0$ and rapidly rotating spin-1 BEC have never been reported in the literature.

The organization of this paper is as follows. In section “Existence of nontrivial solution curves” we present the existence of nontrivial solution curves branching from bifurcation points of the CGPEs. A multi-parameter continuation algorithm is proposed in section “A multilevel continuation algorithm” for computing the ground state of (rapidly) rotating spin-1 BEC. In section “Numerical results” we investigate numerically how the magnetization may affect the behavior of the CGPEs. Our numerical results demonstrate that various vortex lattices of $^{87}Rb$ and $^{23}Na$ can be observed. Finally, some concluding remarks are given in section “Conclusions”.

## Existence of nontrivial solution curves

In this section, we will show the existence of nontrivial solution branches of Eq. (10), where the wave functions near the bifurcation point satisfy $\Vert \psi _{1}\Vert ^2+\Vert \psi _{0}\Vert ^2 +\Vert \psi _{-1}\Vert ^2=O(\varepsilon )$. We consider the scaling $\psi _{l}({\mathbf{x}})=\varepsilon ^{1/2}\phi _l({\mathbf{x}})$, $l=1,0,-1$, Then Eq. (10) becomes

13 $$\begin{aligned} f_1&=  {} \left[ -\frac{\displaystyle 1}{\displaystyle 2}\nabla ^2 +V({\mathbf{x}})-(\mu +\lambda )-\omega L_z\right] \phi _1+\varepsilon \widetilde{N} \left[ g_n(|\phi _1|^2+|\phi _0|^2+|\phi _{-1}|^2)\phi _1\right. \nonumber \\&\quad \left. +g_s(|\phi _1|^2+|\phi _0|^2-|\phi _{-1}|^2)\phi _1 + g_s{\phi }_{-1}^*\phi _0^2\right] =0,\nonumber \\ f_0&=  {} \left[ -\frac{\displaystyle 1}{\displaystyle 2}\nabla ^2 +V({\mathbf{x}})-\mu -\omega L_z\right] \phi _0+\varepsilon \widetilde{N} \left[ g_n(|\phi _1|^2+|\phi _0|^2+|\phi _{-1}|^2)\phi _0\right. \nonumber \\&\quad \left. +g_s(|\phi _1|^2+|\phi _{-1}|^2)\phi _0 + 2g_s\phi _{-1}{\phi }_{0}^*\phi _1\right] =0,\nonumber \\ f_{-1}&=  {} \left[ -\frac{\displaystyle 1}{\displaystyle 2}\nabla ^2 +V({\mathbf{x}})-(\mu -\lambda )-\omega L_z\right] \phi _{-1}+\varepsilon \widetilde{N} \left[ g_n(|\phi _1|^2+|\phi _0|^2+|\phi _{-1}|^2)\phi _{-1}\right. \nonumber \\&\quad \left. +g_s(|\phi _{-1}|^2+|\phi _0|^2-|\phi _{1}|^2)\phi _{-1} + g_s{\phi }_{1}^*\phi _0^2\right] =0, \end{aligned}$$

with $\Vert \phi _{1}\Vert ^2+\Vert \phi _{0}\Vert ^2+\Vert \phi _{-1}\Vert ^2=O(1)$. In order to be consistent with the continuation algorithm we describe in Section 3, we set $\lambda =0$. The linear eigenvalue problem associated with Eq. (13) is given by

14 $$\begin{aligned} -\frac{\displaystyle 1}{\displaystyle 2}\nabla ^2u+V({\mathbf{x}})u -\omega L_z u=\mu u. \end{aligned}$$

For simplicity we let $\omega =0$. The eigenpairs of Eq. (14) are as follows:

15 $$\begin{aligned} \mu _{m,n}&=  {} m+n+1,\ m,n=0,1,2,\dots , \nonumber \\ u_{m,n}&=  {} \frac{\displaystyle 1}{\displaystyle \sqrt{2^{m+n}m!n!\pi }} e^{-\frac{x^2+y^2}{2}}H_m(x)H_n(y), \end{aligned}$$

where $H_k$ is the *k*th degree Hermite polynomial. The first few eigenfunctions are

$$\begin{aligned} u_{0,0}&=  {} \frac{1}{\sqrt{\pi }}e^{-\frac{x^2+y^2}{2}},\ u_{1,0} =\sqrt{\frac{2}{\pi }}\,xe^{-\frac{x^2+y^2}{2}},\ u_{0,1}=\sqrt{\frac{2}{\pi }} \,ye^{-\frac{x^2+y^2}{2}},\\ u_{1,1}&=  {} \frac{2}{\sqrt{\pi }}\,xye^{-\frac{x^2+y^2}{2}},\ \ldots . \end{aligned}$$

Note that the set of eigenfunctions $\left\{ u_{m,n}(x,y)\,|\,m,n =0,1,2, \ldots \right\}$ forms an orthonormal basis for $L^2({\mathbb {R}}^2)$ under the inner product $\left\langle f, g\right\rangle =\int _{{\mathbb {R}}^2} f({\mathbf{x}})g({\mathbf{x}})\,d{\mathbf{x}}.$ Using the Lyapunov–Schmidt reduction it was shown in^58^ that the bifurcations of a single NLS are pitchfork. For the case of BEC the coefficient of the cubic nonlinear term is positive. Thus the pitchfork bifurcations are supercritical where the solution curves turn to right. It is straightforward to prove that the bifurcations of Eq. (10) have the same properties mentioned above. The stability analysis for the CGPES was studied in^61^. It is expected that the stability analysis for spin-1 BEC can be treated in a similar way.

To analyze the existence of the solution branches, we apply the Lyapunov–Schmidt reduction method to Eq. (13) near the bifurcation points, namely $\mu \approx \mu _{m,n}$. The reduction guarantees that $\varvec{\phi }=[\phi _1, \phi _0, \phi _{-1}]^T$ and $\mu$ have the asymptotic expansions in $\varepsilon$, i.e.,

16 $$\begin{aligned} \varvec{\phi } =\varvec{\phi }^{(0)}+\varepsilon \varvec{\phi }^{(1)} + O(\varepsilon ^2) \ \text{ and } \ \mu =\mu ^{(0)}+\varepsilon \mu ^{(1)} + O(\varepsilon ^2). \end{aligned}$$

Moreover, the Fréchet derivative ${\mathcal {L}}$ of the nonlinear functional $f(\varvec{\phi },\varvec{\phi }^*) :=$ $[f_1, f_1^*, f_0, f_0^*,f_{-1}, f_{-1}^*]^T$ at $\varvec{\phi }^{(0)}$ and $\mu ^{(0)}$ also have the asymptotic expansion

17 $$\begin{aligned} {\mathcal {L}}=D f(\varvec{\phi }^{(0)},{\varvec{\phi }^{(0)}}^*) =\left[ \begin{array}{cccccc} D_{\phi _1}f_1 &{}\quad D_{\phi _1^*}f_1 &{}\quad D_{\phi _0}f_1 &{}\quad D_{\phi _0^*}f_1 &{}\quad D_{\phi _{-1}}f_1 &{}\quad D_{\phi _{-1}^*}f_1\\ D_{\phi _1}f_1^*&{}\quad D_{\phi _1^*}f_1^*&{}\quad D_{\phi _0}f_1^*&{}\quad D_{\phi _0^*}f_1^*&{}\quad D_{\phi _{-1}}f_1^*&{}\quad D_{\phi _{-1}^*}f_1^*\\ D_{\phi _1}f_0 &{}\quad D_{\phi _1^*}f_0 &{}\quad D_{\phi _0}f_0 &{}\quad D_{\phi _0^*}f_0 &{}\quad D_{\phi _{-1}}f_0 &{}\quad D_{\phi _{-1}^*}f_0\\ D_{\phi _1}f_0^*&{}\quad D_{\phi _1^*}f_0^*&{}\quad D_{\phi _0}f_0^*&{}\quad D_{\phi _0^*}f_0^*&{}\quad D_{\phi _{-1}}f_0^*&{}\quad D_{\phi _{-1}^*}f_0^*\\ D_{\phi _1}f_{-1} &{}\quad D_{\phi _1^*}f_{-1} &{}\quad D_{\phi _0}f_{-1} &{}\quad D_{\phi _0^*}f_{-1} &{}\quad D_{\phi _{-1}}f_{-1} &{}\quad D_{\phi _{-1}^*}f_{-1}\\ D_{\phi _1}f_{-1}^*&{}\quad D_{\phi _1^*}f_{-1}^*&{}\quad D_{\phi _0}f_{-1}^*&{}\quad D_{\phi _0^*}f_{-1}^*&{}\quad D_{\phi _{-1}}f_{-1}^*&{}\quad D_{\phi _{-1}^*}f_{-1}^*\end{array}\right] ={\mathcal {L}}^{(0)}+\varepsilon {\mathcal {L}}^{(1)}, \end{aligned}$$

where the diagonal terms are

$$\begin{aligned} D_{\phi _1}f_1&=  {} -\frac{\displaystyle 1}{\displaystyle 2}\nabla ^2 +V({\mathbf{x}})-(\mu ^{(0)}+\lambda )-\omega L_z+ \varepsilon \widetilde{N} \left[ (g_n+g_s)(2|\phi _1^{(0)}|^2+|\phi _0^{(0)}|^2)+(g_n-g_s)| \phi _{-1}^{(0)}|^2 \right] ,\\ D_{\phi _1^*}f_1^*&=  {} -\frac{\displaystyle 1}{\displaystyle 2}\nabla ^2+V({\mathbf{x}})-(\mu ^{(0)}+\lambda )-\omega L_z^*+ \varepsilon \widetilde{N} \left[ (g_n+g_s)(2|\phi _1^{(0)}|^2 +|\phi _0^{(0)}|^2)+(g_n-g_s)|\phi _{-1}^{(0)}|^2)\right] ,\\ D_{\phi _0}f_0&=  {} -\frac{\displaystyle 1}{\displaystyle 2}\nabla ^2 +V({\mathbf{x}})-\mu ^{(0)}-\omega L_z+\varepsilon \widetilde{N} \left[ (g_n+g_s)(|\phi _1^{(0)}|^2+|\phi _{-1}^{(0)}|^2) +2g_n|\phi _{0}^{(0)}|^2 \right] ,\\ D_{\phi _0^*}f_0^*&=  {} -\frac{\displaystyle 1}{\displaystyle 2} \nabla ^2+V({\mathbf{x}})-\mu ^{(0)}-\omega L_z^*+\varepsilon \widetilde{N} \left[ (g_n+g_s)(|\phi _1^{(0)}|^2+|\phi _{-1}^{(0)}|^2)+2g_n| \phi _{0}^{(0)}|^2\right] ,\\ D_{\phi _{-1}}f_{-1}&=  {} -\frac{\displaystyle 1}{\displaystyle 2}\nabla ^2 +V({\mathbf{x}})-(\mu ^{(0)}-\lambda )-\omega L_z+\varepsilon \widetilde{N} \left[ (g_n+g_s)(|\phi _0^{(0)}|^2+2|\phi _{-1}^{(0)}|^2)+(g_n-g_s)| \phi _{1}^{(0)}|^2 \right] ,\\ D_{\phi _{-1}^*}f_{-1}^*&=  {} -\frac{\displaystyle 1}{\displaystyle 2} \nabla ^2+V({\mathbf{x}})-(\mu ^{(0)}-\lambda )-\omega L_z^*+\varepsilon \widetilde{N} \left[ (g_n+g_s)(|\phi _0^{(0)}|^2 +2|\phi _{-1}^{(0)}|^2)+(g_n-g_s)|\phi _{-1}^{(0)}|^2)\right] , \end{aligned}$$

and the other terms of ${\mathcal {L}}$ can be computed similarly. Substituting Eq. (16) into Eq. (13), we obtain the systems of equations at *O*(1) and $O(\varepsilon )$, namely,

18 $$\begin{aligned}{}&\left[ -\frac{\displaystyle 1}{\displaystyle 2}\nabla ^2+V({\mathbf{x}}) -(\mu ^{(0)}+\lambda )-\omega L_z\right] \phi _1^{(0)}=0,\nonumber \\&\left[ -\frac{\displaystyle 1}{\displaystyle 2}\nabla ^2+V({\mathbf{x}}) -\mu ^{(0)}-\omega L_z\right] \phi _0^{(0)}=0,\nonumber \\&\left[ -\frac{\displaystyle 1}{\displaystyle 2}\nabla ^2+V({\mathbf{x}}) -(\mu ^{(0)}-\lambda )-\omega L_z\right] \phi _{-1}^{(0)}=0, \end{aligned}$$

and

19 $$\begin{aligned}{}&\left[ -\frac{\displaystyle 1}{\displaystyle 2}\nabla ^2+V({\mathbf{x}}) -(\mu ^{(0)}+\lambda )-\omega L_z\right] \phi _1^{(1)}+ g_s\widetilde{N} {\phi _{-1}^{(0)}}^*(\phi _0^{(0)})^2\nonumber \\&\quad +\left[ \widetilde{N} (g_n+g_s)(|\phi _1^{(0)}|^2 +|\phi _0^{(0)}|^2)+\widetilde{N}(g_n-g_s)|\phi _{-1}^{(0)}|^2 -\mu ^{(1)}\right] \phi _1^{(0)} =0,\nonumber \\&\left[ -\frac{\displaystyle 1}{\displaystyle 2}\nabla ^2+V({\mathbf{x}}) -\mu ^{(0)}-\omega L_z\right] \phi _0^{(1)}+ 2g_s\widetilde{N} \phi _{-1}^{(0)}{\phi _{0}^{(0)}}^*\phi _1^{(0)} \nonumber \\&\quad +\left[ \widetilde{N} (g_n+g_s)(|\phi _1^{(0)}|^2+|\phi _{-1}^{(0)}|^2) +\widetilde{N}g_n|\phi _{0}^{(0)}|^2 -\mu ^{(1)}\right] \phi _0^{(0)}=0,\nonumber \\&\left[ -\frac{\displaystyle 1}{\displaystyle 2}\nabla ^2+V({\mathbf{x}}) -(\mu ^{(0)}-\lambda )-\omega L_z\right] \phi _{-1}^{(1)} +g_s\widetilde{N} {\phi _{1}^{(0)}}^*(\phi _0^{(0)})^2\nonumber \\&\quad +\left[ \widetilde{N} (g_n+g_s)(|\phi _0^{(0)}|^2+|\phi _{-1}^{(0)}|^2) +\widetilde{N}(g_n-g_s)|\phi _{1}^{(0)}|^2 -\mu ^{(1)}\right] \phi _{-1}^{(0)} =0, \end{aligned}$$

respectively. Recall that we choose $\lambda =0$ and $\omega =0$. Then both Eq. (18) and (19) can be simplified. From Eqs. (14) and (18) we have

20 $$\begin{aligned} \varvec{\phi }^{(0)}=\left[ \begin{array}{c} \phi _{1}^{(0)}\\ \phi _{0}^{(0)}\\ \phi _{-1}^{(0)} \end{array}\right] =\left[ \begin{array}{c} au_{m,n}\\ bu_{m,n}\\ cu_{m,n} \end{array}\right] \,\,\text{ and } \,\,\mu ^{(0)}=\mu _{m,n}, \end{aligned}$$

where $a, b, c \in {\mathbb {R}}$ will be determined by Eq. (19). Equation (20) is referred to as the single mode approximation. If

21 $$\begin{aligned}{}&\left\langle \left[ \widetilde{N} (g_n+g_s)(|\phi _1^{(0)}|^2 +|\phi _0^{(0)}|^2)+\widetilde{N}(g_n-g_s)|\phi _{-1}^{(0)}|^2 -\mu ^{(1)}\right] \phi _1^{(0)}+ g_s\widetilde{N} {\phi _{-1}^{(0)}} (\phi _0^{(0)})^2,\ u_{m,n}\right\rangle =0,\nonumber \\&\left\langle \left[ \widetilde{N} (g_n+g_s)(|\phi _1^{(0)}|^2 +|\phi _{-1}^{(0)}|^2)+\widetilde{N}g_n|\phi _{0}^{(0)}|^2 -\mu ^{(1)}\right] \phi _0^{(0)} + 2g_s\widetilde{N} \phi _{-1}^{(0)} \phi _0^{(0)}\phi _1^{(0)},\ u_{m,n}\right\rangle =0,\nonumber \\&\left\langle \left[ \widetilde{N} (g_n+g_s)(|\phi _0^{(0)}|^2 +|\phi _{-1}^{(0)}|^2)+\widetilde{N}(g_n-g_s)|\phi _{1}^{(0)}|^2 -\mu ^{(1)}\right] \phi _{-1}^{(0)}+ g_s\widetilde{N} {\phi _{1}^{(0)}} (\phi _0^{(0)})^2, \,\,u_{m,n}\right\rangle =0, \end{aligned}$$

then Eq. (19) is solvable. Since $\Vert u_{m,n}\Vert ^2=1$, Equation (21) implies that

22 $$\begin{aligned}{}&a \left[ \widetilde{N} (g_n+g_s)A(a^2+b^2)+\widetilde{N}(g_n-g_s)Ac^2 -\mu ^{(1)} \right] + g_s\widetilde{N} Acb^2=0,\nonumber \\&b\left[ \widetilde{N} (g_n+g_s)A(a^2+c^2)+\widetilde{N}g_n Ab^2 -\mu ^{(1)} + 2g_s\widetilde{N}Aac \right] =0,\nonumber \\&c \left[ \widetilde{N} (g_n+g_s)A(b^2+c^2) +\widetilde{N}(g_n-g_s)Aa^2 -\mu ^{(1)}\right] + g_s\widetilde{N}Aab^2 =0, \end{aligned}$$

where $A=A_{m,n}=\left\langle u^2_{m,n},u^2_{m,n}\right\rangle$. The solutions of Eq. (22) have the following five cases:

1. $$a=0, b=0$$, and $$c^2=\displaystyle \frac{\mu ^{(1)}}{(g_n+g_s) \widetilde{N} A}$$  for $$\mu ^{(1)}>0$$;
2. $$a=0, c=0$$, and $$b^2=\displaystyle \frac{\mu ^{(1)}}{g_n\widetilde{N} A}$$  for $$\mu ^{(1)}>0$$;
3. $$b=0, c=0$$, and $$a^2=\displaystyle \frac{\mu ^{(1)}}{(g_n+g_s)\widetilde{N} A}$$  for $$\mu ^{(1)}>0$$;
4. $$b=0$$, and $$a^2=c^2=\displaystyle \frac{ \mu ^{(1)}}{2g_n\widetilde{N} A}$$  for $$\mu ^{(1)}>0$$;
5. $$a, b, c \ne 0$$, and
  $$\begin{aligned} \left\{ \begin{array}{l} (g_n+g_s)(a^2+b^2)+(g_n-g_s)c^2 + g_s \frac{cb^2}{a} =\displaystyle \frac{\mu ^{(1)} }{\widetilde{N} A}, \\ (g_n+g_s)(a^2+c^2)+g_nb^2 + 2g_s ac =\displaystyle \frac{\mu ^{(1)}}{\widetilde{N} A}, \\ (g_n+g_s)(b^2+c^2)+(g_n-g_s)a^2 + g_s \frac{ab^2}{c} =\displaystyle \frac{\mu ^{(1)} }{\widetilde{N} A}. \end{array} \right. \end{aligned}$$

In the first three cases, Eq. (13) reduces to the governing equation for (rotating) one-component BEC. In the fourth case we obtain the governing equations for (rotating) two-component BEC. In Eq. (22), solutions with $a=0, b^2\ne 0, c^2\ne 0$; and $c=0, a^2\ne 0, b^2\ne 0$ do not exist. The last case corresponds the system of governing equations for (rotating) spin-1 BEC.

## A multilevel continuation algorithm

We have the following result.

### Lemma 3.1

*If* $M = \pm 1$, *then Eq.* (10) *reduces to a single GPE.*

### Proof

From Eqs. (6) and (9) we have

$$\begin{aligned} \Vert \Psi \Vert ^2=\Vert \psi _1\Vert ^2+\Vert \psi _0\Vert ^2+\Vert \psi _{-1}\Vert ^2 = 1. \end{aligned}$$

If $M=1$, then from Eq. (7) we have $\Vert \psi _{-1}\Vert =0$ and $\Vert \psi _1\Vert = 1$. Therefore, we obtain $\Vert \psi _0\Vert = 0$, and Eq. (10) reduces to

$$\begin{aligned} (\mu +\lambda )\psi _1 = \left[ -\frac{\displaystyle 1}{\displaystyle 2} \nabla ^2+V({\mathbf{x}})+ (g_n+g_s)\widetilde{N}|\psi _1|^2-\omega L_z\right] \psi _1, \end{aligned}$$

which is a single GPE. The result for $M=-1$ can be proved in a similar way. $\square$

To study numerical solutions of the CGPEs, we replace the whole space ${\mathbb {R}}^2$ in Eq. (10) by a finite domain $\Omega = (-L, L)^2$, where *L* is a positive constant yet to be specified which is large enough. Next, we transform the domain $\Omega$ into $\Omega _1 = (0, 1)^2$ using the change of variables ${\mathbf{x}}=L(2\widetilde{\mathbf{x}}-{\mathbf{1}})$,${\mathbf{1}}= [1,1]^T$, and $\widetilde{\mathbf{x}}\in \Omega _1$. Let $\psi _l({\mathbf{x}}) = u_l({\mathbf{x}})+{\mathbf{i}}v_l({\mathbf{x}})$, $l = -1, 0, 1$ in Eq. (10), where $u_l({\mathbf{x}})$ and $v_l({\mathbf{x}})$ are real-valued functions. We rewrite Eq. (10) as

23 $$\begin{aligned} F(\tilde{\mathbf{v}}, \Lambda ) =\left[ F_1(\tilde{\mathbf{v}}, \Lambda ) , \ldots , F_6(\tilde{\mathbf{v}}, \Lambda ) \right] ^T={\mathbf{0}}, \end{aligned}$$

where $\tilde{{\mathbf{v}}} = (u_1, v_1, u_0, v_0, u_{-1}, v_{-1})^T$, $\Lambda = (\mu , \lambda )$ is the parameter variable, and

24 $$\begin{aligned} F_1(\tilde{\mathbf{v}}, \Lambda )&=  {} \left[ -\frac{\displaystyle 1}{\displaystyle 8L^2}\nabla ^2+\widetilde{V}({\mathbf{x}}) + g_n\widetilde{N}|\Psi |^2-g_s\widetilde{N}(|\psi _1|^2 +|\psi _0|^2-|\psi _{-1}|^2)-(\mu +\lambda )\right] u_1\nonumber \\&\quad -\omega \left( x(v_1)_y-y(v_1)_x\right) + g_s\widetilde{N} (u_0^2u_{-1}-v_0^2u_{-1}+2u_0v_0v_{-1}),\nonumber \\ F_2(\tilde{\mathbf{v}}, \Lambda )&=  {} \left[ -\frac{\displaystyle 1}{\displaystyle 8L^2}\nabla ^2+\widetilde{V}({\mathbf{x}}) + g_n\widetilde{N}|\Psi |^2-g_s\widetilde{N}(|\psi _1|^2 +|\psi _0|^2-|\psi _{-1}|^2)-(\mu +\lambda )\right] v_1\nonumber \\&\quad +\omega \left( x(u_1)_y-y(u_1)_x\right) + g_s\widetilde{N} (v_0^2v_{-1}-u_0^2v_{-1}+2u_0v_0u_{-1}),\nonumber \\ F_3(\tilde{\mathbf{v}}, \Lambda )&=  {} \left[ -\frac{\displaystyle 1}{\displaystyle 8L^2}\nabla ^2+\widetilde{V}({\mathbf{x}}) + g_n\widetilde{N}|\Psi |^2+g_s\widetilde{N}(|\psi _1|^2 +|\psi _{-1}|^2)-\mu \right] u_0\nonumber \\&\quad -\omega \left( x(v_0)_y-y(v_0)_x\right) + 2g_s\widetilde{N} \left( u_1(u_0u_{-1} + v_0v_{-1})+v_1(v_0u_{-1}-u_0v_{-1})\right) ,\nonumber \\ F_4(\tilde{\mathbf{v}}, \Lambda )&=  {} \left[ -\frac{\displaystyle 1}{\displaystyle 8L^2}\nabla ^2+\widetilde{V}({\mathbf{x}}) + g_n\widetilde{N}|\Psi |^2+g_s\widetilde{N}(|\psi _1|^2 +|\psi _{-1}|^2)-\mu \right] v_0\nonumber \\&\quad +\omega \left( x(u_0)_y-y(u_0)_x\right) + 2g_s\widetilde{N} \left( u_1(u_0v_{-1}-v_0u_{-1}) + v_1(u_0u_{-1}+v_0v_{-1})\right) ,\nonumber \\ F_5(\tilde{\mathbf{v}}, \Lambda )&=  {} \left[ -\frac{\displaystyle 1}{\displaystyle 8L^2}\nabla ^2+\widetilde{V}({\mathbf{x}}) + g_n\widetilde{N}|\Psi |^2+g_s\widetilde{N}(|\psi _0|^2 +|\psi _{-1}|^2-|\psi _1|^2)-(\mu -\lambda )\right] u_{-1}\nonumber \\&\quad -\omega \left( x(v_{-1})_y-y(v_{-1})_x\right) + g_s\widetilde{N} (u_1u_0^2-u_1v_0^2+2v_1u_0v_0),\nonumber \\ F_6(\tilde{{\mathbf{v}}}, \Lambda )&=  {} \left[ -\frac{\displaystyle 1}{\displaystyle 8L^2}\nabla ^2+\widetilde{V}({\mathbf{x}}) + g_n\widetilde{N}|\Psi |^2+g_s\widetilde{N}(|\psi _0|^2 +|\psi _{-1}|^2-|\psi _1|^2)-(\mu -\lambda )\right] v_{-1}\nonumber \\&\quad +\omega \left( x(u_{-1})_y-y(u_{-1})_x\right) + g_s\widetilde{N} (v_1v_0^2-v_1u_0^2+2u_1u_0v_0), \end{aligned}$$

and the trapping potential is replaced by $\widetilde{V}({\mathbf{x}})$. The Fourier sine functions will be used as the basis functions for the spectral collocation method (SCM)^62^ to discretize Eq. (23). Let

$$\begin{aligned} V^2_N= span\{\sin i\pi x \sin j\pi y \mid i, j=1,2,\ldots , N, \quad x,y\in [0,1]\} \end{aligned}$$

be the trial function space with the uniform grids $\{(x_m, y_n) =(m/(N+1), n/(N+1)), m, n = 1, 2, \ldots , N\}$ as the collocation points. All functions of $V^2_N$ satisfy the boundary condition $\psi _l|_{\partial \Omega _1}=0$, $l = -1, 0, 1$. The SCM for Eq. (23) is to find the approximate solutions $u_{j}^{N}(x,y)$ and $v_{j}^{N}(x,y)$, for the wave function $\Psi =(\psi _{1},\psi _{0},\psi _{-1}), j = 1, 0,-1,$ where

25 $$\begin{aligned} u_1^N(x,y) = \sum \limits _{i,j=1}^{N}\alpha _{i,j}\sin i\pi x\sin j\pi y, v_1^N(x,y) = \sum \limits _{i,j=1}^{N}\beta _{i,j}\sin i\pi x\sin j\pi y \,\in V^2_N, \end{aligned}$$

and $u_{0}^{N}$, $u_{-1}^{N}$, $v_{0}^{N}$, $v_{-1}^{N}$ can be expressed in a similar way except that the coefficients are replaced by $\widetilde{\alpha }_{i,j}$, $\widehat{\alpha }_{i,j}$, $\widetilde{\beta }_{i,j}$, $\widehat{\beta }_{i,j}$, respectively. Note that the residuals vanish at the collocation points, i.e.,

$$\begin{aligned}{}&F_{j}\left( u_{1}^{N}(x_m, y_n),v_{1}^{N}(x_m, y_n),u_{0}^{N}(x_{m}, y_n{}), v_{0}^{N}(x_{m}, y_{n}),u_{-1}^{N}(x_{m}, y_{n}),v_{-1}^{N}(x_{m}, y_{n}),\mu ,\lambda \right) =0,\\&\quad j=1,\dots , 6,\ m, n = 1, 2,\dots , N. \end{aligned}$$

The nonlinear system associated with Eq. (24) can be expressed as

26 $$\begin{aligned} H(\alpha ,\beta ,\widetilde{\alpha },\widetilde{\beta },\widehat{\alpha }, \widehat{\beta },\mu ,\lambda ):= [H_{1}(\tilde{\mathbf{x}},\Lambda ),H_{2} (\tilde{\mathbf{x}},\Lambda ),\ldots ,H_{6}(\tilde{\mathbf{x}},\Lambda )]^T. \end{aligned}$$

Here $$H:{\mathbb {R}}^{6N^2+2}\rightarrow {\mathbb {R}}^{6N^2}$$ is a smooth mapping with the components $$H_j, j = 1, 2,\ldots , 6$$, and $$\tilde{\mathbf{x}} =(\alpha ,\beta ,\widetilde{\alpha }, \widetilde{\beta },\widehat{\alpha },\widehat{\beta })$$ is the state variable yet to be determined, whose components are the vectors associated with the coefficients of $$u_{j}^{N}(x,y)$$ and $$v_{j}^{N}(x,y), j = 1, 0, -1$$, say, $$\alpha =[\alpha _{1,1}, \ldots ,\alpha 
_{1,N},\ldots ,\alpha _{N,1},\ldots ,\alpha _{N,N}]^T$$, and so on. We denote the Jacobian matrix associated with *H* by $$DH\in {\mathbb {R}}^{(6N^2)\times (6N^2+2)}$$.

Various types of continuation algorithms have been proposed for computing the ground state and excited states of (rotating) BEC^60,62–64^. In this section, we describe a multi-parameter continuation algorithm for computing the ground state solution of Eq. (10). It suffices to trace the solution curve branching from the minimum eigenvalue of the linearized Schrödinger equation (LSE) associated with Eq. (10). Starting with $\Vert \Psi \Vert \approx 0$ near the trivial solution curve, we will follow this primary solution curve by the proposed continuation algorithm described below until the target point is reached, where the normalization $\Vert \Psi \Vert ^2 = 1$ is satisfied. See Eq. (6). The target point we obtained is indeed the ground state solution of Eq. (10). A detailed comparison between the performance of the PITEM/CNGF and continuation methods was reported in^36^. We also refer to^60,61^ for further discussions. Furthermore, we will obtain all solutions of rotating spin-1 BEC for any values of the particle number $\widetilde{N}$ (or the angular velocity $\omega$) on certain interval, say, $\widetilde{N} \in [\widetilde{N}_0, \widetilde{N}^*]$ for some positive number $\widetilde{N}^*$ (or $\omega \in [\omega _0, \omega ^*]$). Note that in some cases the ground state solution of the NLS does not necessarily lie on the nontrivial solution curve branching from the minimum eigenvalue of the associated LSE. See e.g.,^65,66^.

Theoretically we can use both the chemical potential $\mu$ and the parameter $\widetilde{N}$ as the two continuation parameters simultaneously. However, the continuation increment, namely, the step size for curve-tracing is relatively small, say, from $10^{-1}$ to $10^{-2}$, depending on the curvature of the solution curve, compared to the scale of $\widetilde{N}$. Therefore, it requires large number of continuation steps to trace the solution curve which can be very expensive. To overcome the drawback, we impose an additional parameter $\nu \in [0,\nu ^*]$ and set $\widetilde{N}=\widetilde{N}_0+\nu \widetilde{\sigma }$ in Eq. (23) for some constants $\widetilde{N}_0$ and $\widetilde{\sigma }$. Thus, the first component of Eq. (23) can be expressed as

$$\begin{aligned} F_1(\tilde{\mathbf{v}}, \Lambda )&=  {} \left[ -\frac{\displaystyle 1}{\displaystyle 8L^2}\nabla ^2+\widetilde{V}({\mathbf{x}}) + g_n(\widetilde{N}_0+\nu \widetilde{\sigma })|\Psi |^2 - g_s(\widetilde{N}_0+\nu \widetilde{\sigma })(|\psi _1|^2 +|\psi _0|^2-|\psi _{-1}|^2)\right] u_1\\&\quad -\omega \left( x(v_1)_y-y(v_1)_x\right) + g_s(\widetilde{N}_0+\nu \widetilde{\sigma })(u_0^2u_{-1} -v_0^2u_{-1}+2u_0v_0v_{-1})-(\mu +\lambda )u_1=0, \end{aligned}$$

where the parameter variable $\Lambda =(\mu ,\lambda )$ is updated to $\Lambda =(\mu ,\lambda ,\nu )$. Other components of Eq. (23) can be expressed in a similar way. For convenience we also refer to this expression as Eq. (23).

To begin with, we consider the first level continuation algorithm with $\Lambda = \mu \in {\mathbb {R}}$ as the continuation parameter, and set $\lambda = \nu = 0$. The discrete analogue of Eq. (23) is a nonlinear system of equations involving the parameter $\mu$, and is given as

27 $$\begin{aligned} H(\alpha ,\beta ,\widetilde{\alpha },\widetilde{\beta },\widehat{\alpha }, \widehat{\beta },\mu )= H(\tilde{\mathbf{x}},\Lambda ) =\left[ H_1(\tilde{\mathbf{x}},\Lambda ),H_2(\tilde{\mathbf{x}},\Lambda ), \ldots , H_6(\tilde{\mathbf{x}},\Lambda )\right] ^T={\mathbf{0}}. \end{aligned}$$

We denote a solution curve of Eq. (27) by

$$\begin{aligned} c=\left\{ {\mathbf{y}}(s)=(\alpha (s),\beta (s),\widetilde{\alpha }(s), \widetilde{\beta }(s),\widehat{\alpha }(s),\widehat{\beta }(s), \mu (s))\mid H({\mathbf{y}}(s))={\mathbf{0}},s\in I \subset {\mathbb {R}}\right\} . \end{aligned}$$

Assume that a parametrization via arc-length is available on the solution curve *c*. Thus a unit tangent vector $\dot{\mathbf{y}}(s)$ always exists on each point ${\mathbf{y}}(s)$. See^67^ and the further references cited therein. To compute the ground state solution, we start with an initial point ${\mathbf{y}}_1=({\mathbf{0}},{\mathbf{0}},{\mathbf{0}},{\mathbf{0}},{\mathbf{0}},{\mathbf{0}},\mu _1)$ on the trivial solution curve $\{({\mathbf{0}},{\mathbf{0}},{\mathbf{0}},{\mathbf{0}},{\mathbf{0}},{\mathbf{0}},\mu )\mid \mu \in {\mathbb {R}}\}$, where $\mu _1$ is close enough to the minimum eigenvalue of the linear Schrödinger equation (LSE) associated with Eq. (27). Differentiating $H({\mathbf{y}}(s))={\mathbf{0}}$ with respect to the variable *s*, we obtain

28 $$\begin{aligned} DH({\mathbf{y}}(s))\cdot \dot{\mathbf{y}}(s)={\mathbf{0}}, \end{aligned}$$

where the tangent vector $\dot{\mathbf{y}}(s)$ is normalized so that

$$\begin{aligned} \Vert \dot{\mathbf{y}}(s)\Vert = \Vert \left( \dot{\alpha }(s), \dot{\beta }(s), \dot{\widetilde{\alpha }}(s),\dot{\widetilde{\beta }}(s), \dot{\widehat{\alpha }}(s),\dot{\widehat{\beta }}(s), \dot{\mu }(s) \right) \Vert = 1, \end{aligned}$$

and the Jacobian matrix $DH({\mathbf{y}}(s))\in {\mathbb {R}}^{(6N^2) \times (6N^2+1)}$ is of full rank. It follows from Eq. (28) that the augmented Jacobian matrix

$$\begin{aligned} A({\mathbf{y}}(s))= \left[ \begin{array}{c} DH({\mathbf{y}}(s))\\ \dot{{\mathbf{y}}}(s)^T \end{array}\right] \in {\mathbb {R}}^{(6N^2+1)\times (6N^2+1)} \end{aligned}$$

is nonsingular for all $s \in I$ except that at the primary bifurcation points on the trivial solution curve $\{({\mathbf{0}},{\mathbf{0}},{\mathbf{0}},{\mathbf{0}},{\mathbf{0}},{\mathbf{0}},\mu )\,|\,\mu \in {\mathbb {R}}\}$, where the Jacobian matrix $DH({\mathbf{y}}(s))$ has rank deficiency. To switch from the trivial solution curve to the primary solution branch near the bifurcation point, we solve the perturbed nonlinear system

29 $$\begin{aligned} H(\tilde{\mathbf{x}},\Lambda )+ {\mathbf{d}} ={\mathbf{0}} \end{aligned}$$

for some perturbation vector ${\mathbf{d}}\in {\mathbb {R}}^{6N^2}$. In general, the vector ${\mathbf{d}}$ in Eq. (29) is chosen so that it has the same mode as the eigenfunction of the associated linear eigenvalue problem. We refer to^68^ and the further reference cited therein for details.

Right after we switch from the trivial solution curve to the primary solution curve, we perform the second level continuation algorithm by adding the magnetic potential $\lambda$ as the second component of the parameter variable $\Lambda$ defined in Eq. (27). That is, we set $\Lambda _2 :\,=(\mu , \lambda )\in {\mathbb {R}}^{2}$, where the value of the wave function $\Vert \Psi \Vert ^2 \approx k_0$ is small enough for some positive constant $k_0$. Note that if $k_0$ is too large, which means that we implement the first level continuation algorithm to trace the solution curve by neglecting the affect of the magnetic potential $\lambda$. The price is that the algorithm can not mimic the physical systems of rotating spin-1 BEC precisely. This would make the algorithm either diverge or fail to trace the solution curve we wish to follow. We refer to^39^ for detailed discussions. Now we rewrite the magnetization (7) as

30 $$\begin{aligned} H_7(\tilde{\mathbf{x}},\Lambda ) = \int _{{\mathbb {R}}^2}\left[ |\psi _1 (\tilde{\mathbf{x}})|^2-|\psi _{-1}(\tilde{\mathbf{x}})|^2\right] d \tilde{\mathbf{x}}-M\Vert \Psi \Vert ^2 = 0, \end{aligned}$$

which is added as the last equation to the nonlinear system of equations $H(\tilde{\mathbf{x}},\Lambda )={\mathbf{0}}$ defined in Eq. (27). More precisely, we update $H(\tilde{\mathbf{x}}, \Lambda )={\mathbf{0}}$ by setting

31 $$\begin{aligned} \widetilde{H}(\tilde{\mathbf{x}},\Lambda _2)&=  {} \left[ H_1(\tilde{\mathbf{x}}, \Lambda _2), H_2(\tilde{\mathbf{x}},\Lambda _2), \ldots , H_7(\tilde{\mathbf{x}},\Lambda _2)\right] ^T ={\mathbf{0}}. \end{aligned}$$

Now the second level continuation is exploited to trace the ground state solution curve of Eq. (31). We stop the implementation of the second level algorithm when the normalization condition $\Vert \Psi \Vert ^2 = 1$ is satisfied.

Finally, we update the parameter variable $\Lambda _2=(\mu ,\lambda )$ to a three-component variable $\Lambda _3=(\mu ,\lambda ,\nu ) \in {\mathbb {R}}^{3}$, where the last component of $\Lambda _3$ is defined in Eq. (23). We express the normalization condition (6) as

32 $$\begin{aligned} H_8(\tilde{\mathbf{x}},\Lambda ) = \int _{{\mathbb {R}}^2}\sum ^1_{l=-1}|\psi _l (\tilde{\mathbf{x}})|^2d{\tilde{\mathbf{x}}} - 1 = 0. \end{aligned}$$

Equation (32) will be added as the last equation to the nonlinear system of equations $\tilde{H}(\tilde{\mathbf{x}}, \Lambda _2)={\mathbf{0}}$ defined in Eq. (31). In other words, the nonlinear system $\tilde{H}(\tilde{\mathbf{x}},\Lambda _2)={\mathbf{0}}$ will be updated to a new one, namely,

33 $$\begin{aligned} \widehat{H}(\tilde{\mathbf{x}},\Lambda _3)= \left[ H_1(\tilde{\mathbf{x}}, \Lambda _3), H_2(\tilde{\mathbf{x}},\Lambda _3), \ldots , H_8 (\tilde{\mathbf{x}},\Lambda _3)\right] ^T ={\mathbf{0}}. \end{aligned}$$

Denote the Jacobian matrix of $\widehat{H}$ by $D\widehat{H} \in {\mathbb {R}}^{(6N^2+2)\times (6N^2+3)}$. We implement the third level algorithm to trace the ground state solution of Eq. (33). To compute the unit tangent vector $\dot{\mathbf{y}}_{(k)} =(\dot{\alpha }_{(k)},\dot{\beta }_{(k)}, \dot{\widetilde{\alpha }}_{(k)},\dot{\widetilde{\beta }}_{(k)}, \dot{\widehat{\alpha }}_{(k)},\dot{\widehat{\beta }}_{(k)}, \dot{\mu }_{(k)},\dot{\lambda }_{(k)},\dot{\nu }_{(k)}) \in {\mathbb {R}}^{(6N^2+3)}$, we solve the linear system

$$\begin{aligned} \left[ \begin{array}{c} D\widehat{H}(\mathbf{y}_{(k)})\\ {(\dot{\mathbf{y}}_{(k-1)})^T} \end{array}\right] \dot{\mathbf{y}}_{(k)} =\left[ \begin{array}{c} \bar{0} \\ 1 \end{array}\right] , \quad \text{ where } \bar{0}\in {\mathbb {R}}^{6N^2+2}. \end{aligned}$$

A new approximating point is predicted by the Euler predictor

$$\begin{aligned} {\mathbf{z}}_{(k+1,1)} = {\mathbf{y}}_{(k)}+\delta _{(k)}\cdot \dot{\mathbf{y}}_{(k)}, \end{aligned}$$

where $\delta _{(k)} > 0$ is the step length. Next, we correct the predicted point ${\mathbf{z}}_{(k+1,1)}$ by performing Newton’s iteration. We solve the linear system

$$\begin{aligned} \left[ \begin{array}{c} D\widehat{H}({\mathbf{z}}_{(k+1,j)})\\ {(\dot{\mathbf{y}}_{(k)})^T} \end{array}\right] {\varvec{w}}_{(j)} =\left[ \begin{array}{c} -\widehat{H}({\mathbf{z}}_{(k+1,j)}) \\ 0 \end{array}\right] , \quad j=1,2,\ldots , \end{aligned}$$

where ${\mathbf{z}}_{(k+1,j+1)}={\mathbf{z}}_{(k+1,j)}+\varvec{w}_{(j)}$, $j=1,2,\ldots$. If the corrector increment $\Vert \varvec{w}_{(j_0)}\Vert$ and $\Vert \widehat{H}({\mathbf{z}}_{(k+1,j_0+1)})\Vert$ are small enough for some $j_0\in {\mathbb {N}}$, we obtain the next approximating point ${\mathbf{y}}_{(k+1)}={\mathbf{z}}_{(k+1,j_0+1)}$. We stop the curve-tracing when the target point is reached. Now the ground state solutions for various values of the coefficient $\widetilde{N}$ are available on the solution curve. Note that the state variables of Eqs. (27), (31), and (33) are the same. More precisely, the solution curves connect consecutively except that we would gain more information for the ground states as the number of components of the parameter variable increases.

The algorithm described above may be briefly summarized as follows.

### Algorithm 3.2

**A multi-level continuation method for rapidly rotating spin-1 BEC.**

*Initialization*: $k_0 :=$ *a given small positive number for implementing Level 2*.
  $\widetilde{N}_0 :=$ *initial particle number used in Levels 1 and 2*.
  $$M :=$$ *given*.
*Level 1*.: *Set* $\lambda =0$ *and* $\Lambda := (\mu )$. *Implement the first level continuation algorithm to trace the ground state solution until* $\Vert \Psi \Vert ^2\approx k_0$.
*Level 2*.: *Set* $\Lambda := (\mu , \lambda )\in {\mathbb {R}}^2$. *Implement the second level continuation algorithm under the constraint* $\Vert \psi _1\Vert ^2-\Vert \psi _{-1}\Vert ^2 = M\Vert \Psi \Vert ^2$ *until the normalization* $\Vert \Psi \Vert ^2 = 1$ *is reached.*
*Level 3.*: *Set* $\Lambda := (\mu ,\lambda ,\nu )\in {\mathbb {R}}^3$. *Implement the third level continuation algorithm under the constraint* $\Vert \Psi \Vert ^2 = 1$. *Stop the curve-tracing when* $\nu = \nu ^*, { i.e.}, \widetilde{N} = \widetilde{N}^*$ *is reached.*

Table 1 lists the parameters and the associated constraints used in Algorithm 3.2, and the counterparts used in^40^.

### Remark

For tracing the ground state solution curve of ultrarapidly rotating spin-1 BEC, we use the angular velocity $\omega$ as the third components of the parameter variable by letting $\omega =\omega _0 +\nu \widetilde{\sigma }$ in Eq. (24) for some constants $\omega _0$ and $\widetilde{\sigma }$. That is, $\omega \in [\omega _0, \omega ^*]$.

**Table 1:** Comparison of the parameters and the associated constraints between the algorithms for spin-1 BEC and rotating spin-1 BEC.

	Level 1	Level 2	Level 3
**(a) Spin-1 BEC**			
Parameter	$\mu$	$\lambda$	$M\in [0,1]$
Constraint	Unit tangent	Magnetization $M=0$ until $\Vert \Psi \Vert ^2 = 1$	Normalization $\Vert \Psi \Vert ^2 = 1$
**(b) Rotating spin-1 BEC**			
Parameter	$\mu$	$\lambda$, *M*: given	$\nu \leftrightarrow \widetilde{N}$, $\nu \in [0,\nu ^*]$
Constraint	Unit tangent	$\Vert \psi _1\Vert ^2 -\Vert \psi _{-1}\Vert ^2 = M\Vert \Psi \Vert ^2$ until $\Vert \Psi \Vert ^2 = 1$	Normalization $\Vert \Psi \Vert ^2 = 1$

## Numerical results

In this section we report the implementation results of Algorithm 3.2. The ratio of spin-independent and spin-dependent interactions is $\sim {0.48}\%$^69^ for $^{87}Rb$, $\sim {1.5}\%$^70^ for $^{23}Na$, and strongly ferromagnetic effect $\sim {45}\%$^5,71^ for $^{17}Li$. For typical parameters the character length of trap $a_{m}\sim {1}\mu$m and s-wave scattering length $\sim {10}$nm. It has been satisfied for most experiments. By using $a_{m}$ as the length unit, for the ferromagnetic case $^{87}Rb$ we chose $g_n = 0.0885$, and $g_s =-0.00041$, and for the antiferromagnetic case $^{23}Na$ we chose $g_n = 0.0241$, and $g_s =0.00075$. From^72,73^, we have $-0.67<\widetilde{N}a_0($or $a_2)/a_m<\infty$. The particle number of condensate is typically between $10^4$ and $10^6$. In these cases, the GPE model is valid under the dilute condition. Specifically, in Example 4.1 we studied the convergence behavior of Algorithm 3.2 combined with the Fourier sine functions, where the 1D case of Eq. (10) was used as the test problem. In Examples 4.2 and 4.3 we investigated the ground state solutions of Eq. (10) for $^{87}Rb$ and $^{23}Na$ with various number of particles. In Examples 4.4 and 4.5 we studied the ground states solution of rapidly rotating spin-1 BEC for $^{87}Rb$ and $^{23}Na$, where the angular velocity $\omega > 1$ was treated as one of the continuation parameters, and the number of particles $\widetilde{N}$ is fixed. In these two examples we chose $M > 0$ to emphasize how the magnetization may affect the interactions among the three components. Besides, the numerical results of these two examples demonstrated different phase of the ground state solution with respect to the angular velocity. That is, the uniqueness of the ground state holds modulo rotational equivariance.

### Example 4.1

**Figure 1:**
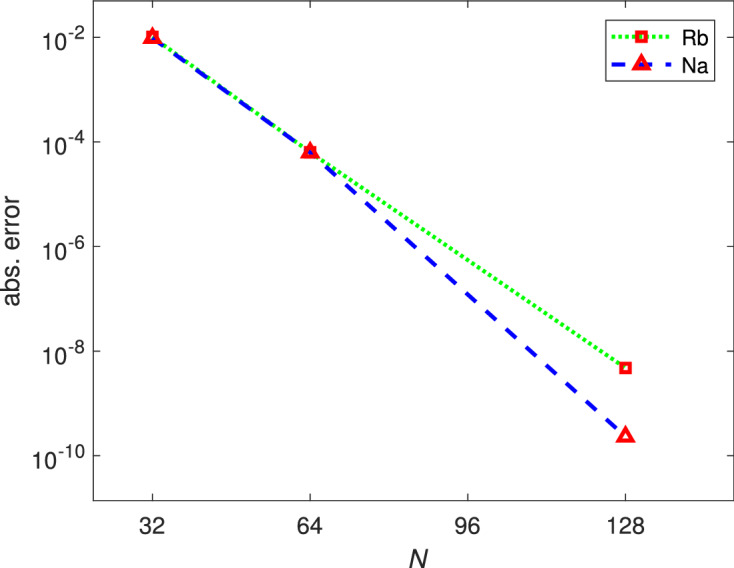
Convergence behavior for the chemical potentials of $^{87}Rb$ and $^{23}Na$.

**Figure 2:**
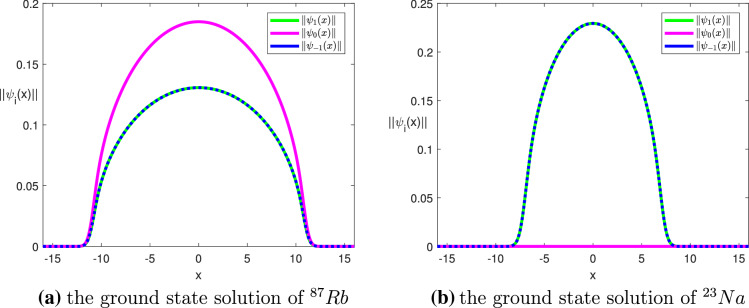
Implementing Levels 1 and 2 of Algorithm 3.2 with $k_0 = 0.0005$ for $^{87}Rb$ and $k_0 =0.01$ for $^{23}Na$, where $M = 0$, $\widetilde{N} = 10^4$, $N=1024$, and $\Omega = (-16,16)$.

(Convergence behavior of Algorithm 3.2 combined with the Fourier sine functions) For simplicity we studied the 1D case of Eq. (10), where we chose $V(x)=x^2/2$, $\omega =0$, $M=0$, $\widetilde{N}=10^4$, and the domain $\Omega = (-16,16)$. We traced the ground state solution curve of spin-1 BEC using $^{87}Rb$ and $^{23}Na$ for different number of basis functions until the normalization condition $\Vert \Psi \Vert ^2=1$ was satisfied. Denote the corresponding chemical potentials and exact chemical potential by $\mu ^{(N)}$, and by $\mu ^*$, respectively, and the convergence rate and convergence order for $^{87}Rb$ or $^{23}Na$ by $e^{-mN}$ and $N^{-Order}$, respectively^74^, which are given by

$$\begin{aligned} |\mu ^{(N)}-\mu ^*| \approx O(e^{-mN})\,\, \Rightarrow \,\, m = \frac{2}{N}\ln \left( \frac{|\mu ^{({N\over 2})}-\mu ^*|}{|\mu ^{(N)}-\mu ^*|}\right) , \end{aligned}$$

and

$$\begin{aligned} |\mu ^{(N)}-\mu ^*| \approx O(N^{-\textit{Order}})\,\, \Rightarrow \,\,\textit{Order} = \log _2\left( \frac{|\mu ^{({N\over 2})}-\mu ^*|}{|\mu ^{(N)}-\mu ^*|}\right) , \end{aligned}$$

respectively. Tables 2–3 list the chemical potentials, the corresponding absolute errors, and the values of *m* and *Order* associated with the convergence behavior of $^{87}Rb$ and $^{23}Na$, respectively. Figure 1 displays the convergence behavior of the chemical potentials for $^{87}Rb$ and $^{23}Na$. The results given above show that the convergence rate of Algorithm 3.2 combined with the Fourier sine functions for the CGPEs, a nonlinear elliptic eigenvalue problem, is indeed exponential. Figure 2a,b show the graphs of the ground state solution of $^{87}Rb$ and $^{23}Na$, respectively, with $N=1024$.

### Example 4.2

**Figure 3:**
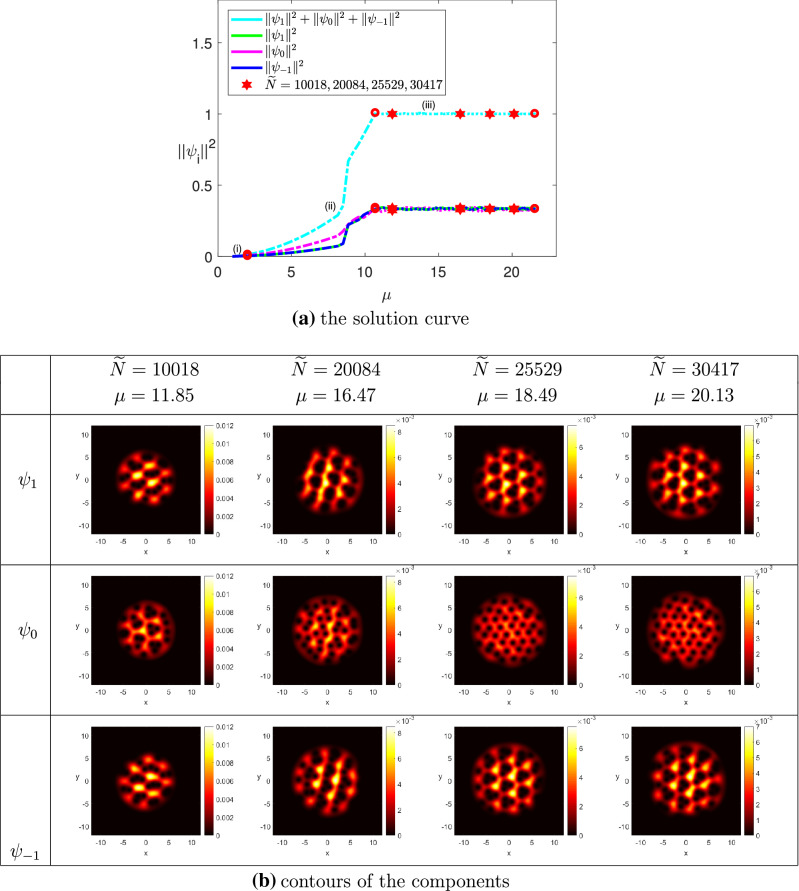
The ground state solutions of $^{87}Rb$, where $k_0 = 0.01$, $M=0$, $\omega =0.75$, and $\widetilde{N}^* = 35{,}000$.

**Figure 4:**
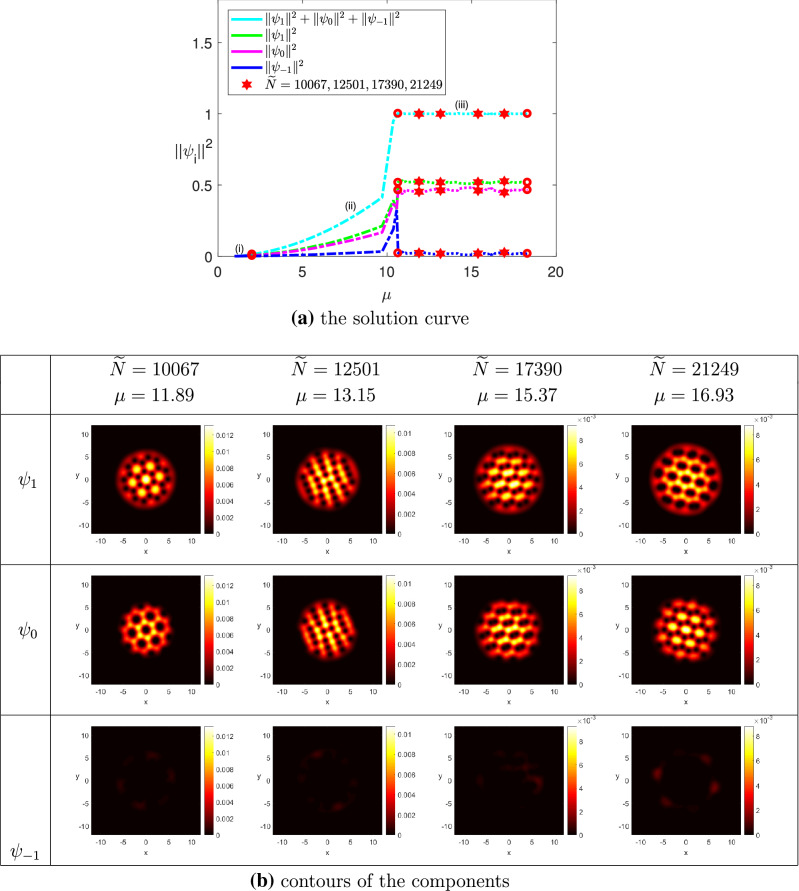
The ground state solutions of $^{87}Rb$, where $k_0 = 0.01$, $M=0.5$, $\omega =0.75$, and $\widetilde{N}^* = 25{,}000$.

(The ground state solutions of rotating spin-1 BEC for $^{{87}}$Rb) We chose $\omega = 0.75$, $N = 50$, $k_0 = 0.01$ and $\Omega = (-12,12)^2$. The minimum eigenvalue of the LSE was detected at $\mu _1 \approx 1$.

(i) $M = 0$: We set $\widetilde{N}_0 = 8000$, $\widetilde{N}^* = 35{,}000$, and $\widetilde{\sigma } = 200$. Figure 3a shows the ground state solution curve of the wave function $\Psi$ together with its projections on the three components using the squares of the two-norm with respect to the chemical potential $\mu$, where the portions (i) and (ii) were obtained by implementing Levels 1 and 2, respectively. The horizontal line (iii) was obtained by implementing Level 3 of Algorithm 3.2, where we added $\nu$ as the third component of the parameter variable, and traced the solution branch until $\nu = 135$, or equivalently, $\widetilde{N}^*= 35{,}000$ was reached. At this step we obtained the contours for $\widetilde{N} \in [8000, 35{,}000]$. Note that the projections on the components $\psi _1$ and $\psi _{-1}$ coincided each other in Levels 1 and 2. Moreover, those of the three components coincided with one another in Level 3. Figure 3b shows that three vortices of the components $\psi _1$ and $\psi _{-1}$ were pinned together to form strip lines or a triangular lattice, while the vortices of the component $\psi _0$ formed a triangular lattice, where $\widetilde{N} \ge 25{,}529$. Our result is quite similar to that in^19^ but not exactly the same because of using different governing equations and coefficients.
(ii) $M = 0.5$: We set $\widetilde{N}_0 = 8000$, $\widetilde{N}^* = 25{,}000$, and $\widetilde{\sigma } = 100$. Figure 4a depicts the ground state solution curve of the wave function $\Psi$ and its projections on the components using the squares of the two-norm with respect to the chemical potential $\mu$. The portions (i), (ii) and (iii) of the solution curve were obtained by implementing Levels 1, 2, and 3 of Algorithm 3.2, respectively. The portion (iii) represents the ground state solutions of the CGPEs under the normalization condition (6) and the magnetization condition (7) simultaneously for all $\widetilde{N} \in [8000,25{,}000]$, i.e., $\nu \in [0,170]$. From Fig. 4b we observed that when $\widetilde{N} = 10{,}067$, a honeycomb lattice was formed for the first component $\psi _1$, while vortices in the second component $\psi _0$ formed a hexagonal droplet lattice, and when $\widetilde{N} = 12{,}501$, the vortex lattices of both $\psi _1$ and $\psi _0$ exhibit strip lines. As $\widetilde{N} = 17{,}390$, two vortices of the two components $\psi _1$ and $\psi _0$ in the lattice were pinned together to form a vortex-pair lattice, where each pair has the same circulation. When $\widetilde{N} = 21249$, the vortex lattice of the first component became a hexagonal droplet lattice, and a honeycomb lattice was formed for the second component $\psi _0$. Moreover, when $\widetilde{N} = 12{,}501$ and 21, 249, vortices in $\psi _1$ were filled by the peaks of $\psi _0$. Similar phenomenon has been observed in some published literature. See e.g.,^1,19,46,47,75,76^. In addition, the two-norm of the third component $\psi _{-1}$ almost equals to zero for all values of $\widetilde{N}$ because of the magnetization $M = 0.5$. It is expected that if we increase the value of magnetization from $M=0.5$ gradually, the two-norm of the third component $\psi _{-1}$ will be zero, and the three-coupled GPEs reduce to rotating two-component BEC. Our result is similar to that of rotating two-component BEC shown in^23^. The result verifies the prediction numerically shown in Lemma 3.1. That is, as the magnetization *M* increases, the governing Eq. (10) will change gradually from the two-coupled GPEs, and then to the single GPE.

### Example 4.3

**Figure 5:**
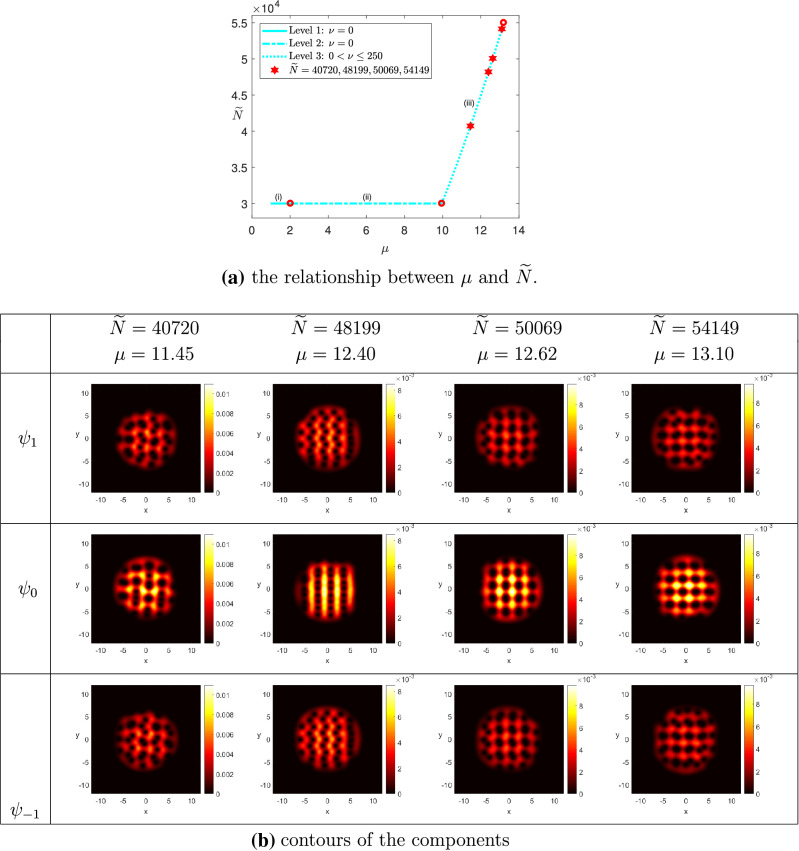
The ground state solutions of $^{23}Na$, where $k_0 = 0.01$, $M=0$, $\omega =0.8$, and $\widetilde{N}^* = 55{,}000$.

**Figure 6:**
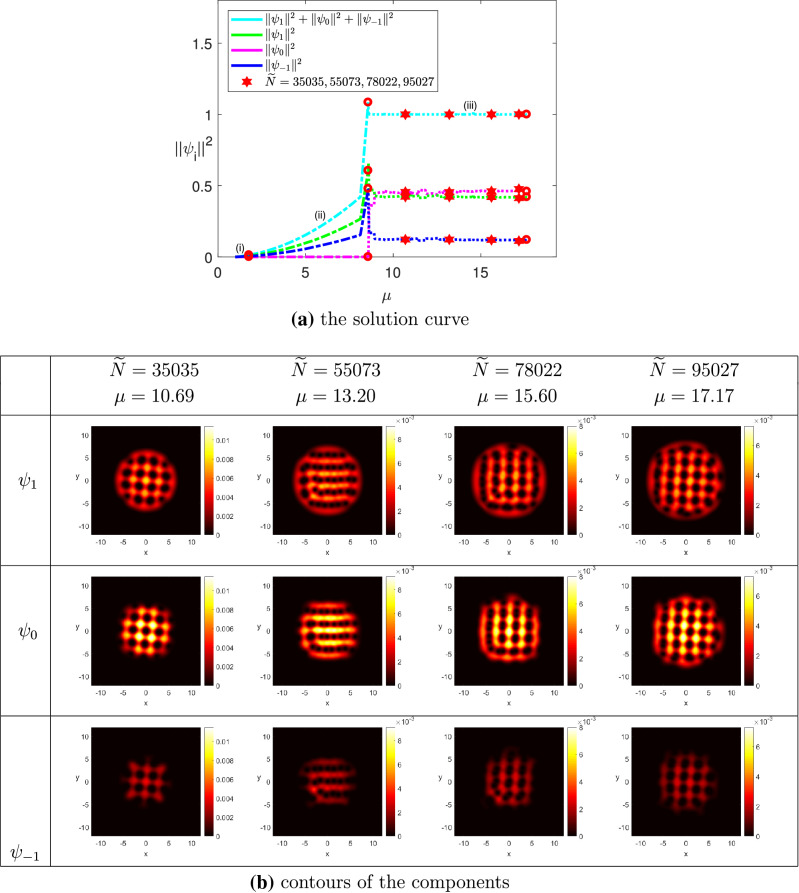
The ground state solutions of $^{23}Na$, where $k_0 = 0.01$, $M=0.3$, $\omega =0.8$, and $\widetilde{N}^* = 100{,}000$.

(The ground state solutions of rotating spin-1 BEC for $^{{23}}$Na) We chose $\omega = 0.8$, $\widetilde{\sigma } = 100$, $N = 50$, $k_0 = 0.01$ and $\Omega = (-12,12)^2$. The minimum eigenvalue of the LSE was detected at $\mu _1 \approx 1$.

(i) $M = 0$: We set $\widetilde{N}_0 = 30{,}000$, and $\widetilde{N}^* = 55{,}000$. Figure 5a shows the relationship between the chemical potential $\mu$ and the particle numbers $\widetilde{N}$ on the two-norm solution curve of the wave function $\Psi$ obtained in implementing Levels 1–3. Figure 5b displays how the vortex lattice of the components evolve with respect to the particle number $\widetilde{N}$. More precisely, when $\widetilde{N} \approx 40{,}720$, two vortices of the components $\psi _{1}$ and $\psi _{-1}$ start to be pinned together. When $\widetilde{N} \approx 48{,}199$, vortices in the three component were pinned together to form a vortex-pair lattice, where the vortices of each pair had the same circulation. When the particle number is large enough, say, $\widetilde{N} \ge 50{,}069$, the vortices of the three components exhibit a square lattice where two vortices remained to be pinned together. Similar phenomenon has been observed in published literature on rotating spin-1 BEC. See e.g.,^19^.
(ii) $M = 0.3$: We set $\widetilde{N}_0 = 20{,}000$, and $\widetilde{N}^* = 100{,}000$. Algorithm 3.2 was implemented to compute the ground state solution curve of the wave function $\Psi$. The result was depicted in Fig. 6a, which showed that the projection on the second component $\psi _0$ equals zero in Levels 1 and 2, and then increases slowly in Level 3. On the other hand, the projections on the components $\psi _1$ and $\psi _{-1}$ increase in Levels 1 and 2, and decrease in Level 3, which separate from each other owing to the magnetization. Figure 6b displayed how the contour plots of the three components varied with respect to the value of $\widetilde{N}$. When $\widetilde{N} \ge 78{,}022$, we found that the vortices of the three components exhibited a square lattice, which are similar to the case in (i) with $M = 0$. However, the two-norm of $\psi _{-1}$ is smaller than the counterpart of (i).

### Example 4.4

(The ground state solution of ultrarapidly rotating spin-1 BEC for $^{{87}}$Rb) In order to make a stronger confinement on the physical system, we replaced the harmonic trapping potential in Eq. (10) by the harmonic plus quartic one which has the following form

$$\begin{aligned} V({\mathbf{x}})=(x^2+y^2)/2+(x^2+y^2)^2/4. \end{aligned}$$

**Figure 7:**
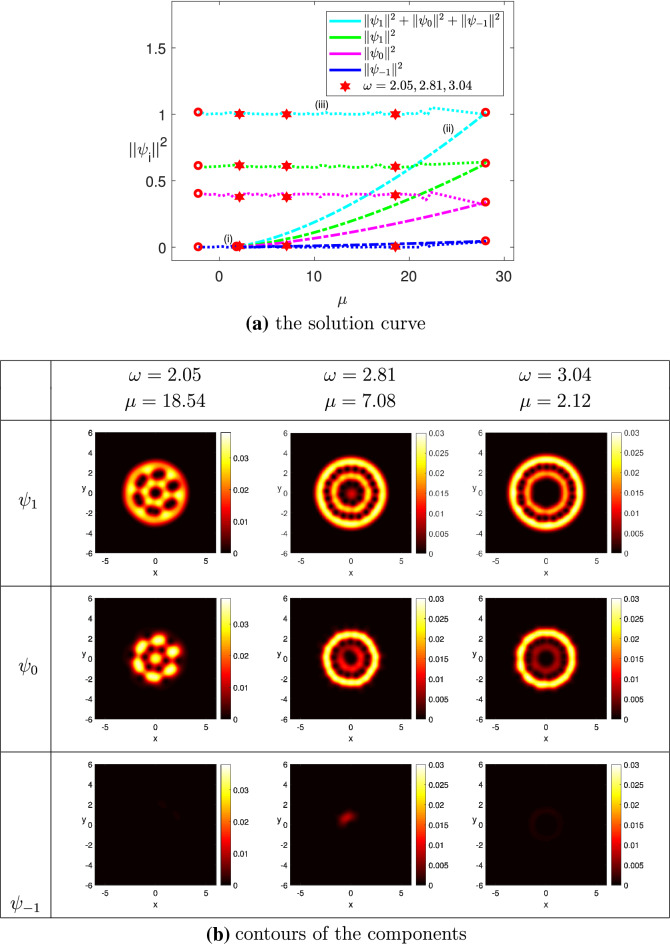
The ground state solution of $^{87}Rb$, where $k_0 = 0.01$, $M=0.6$, $\widetilde{N} = 6000$, and $\omega \in [0.95, 3.30]$.

The angular velocity $\omega$ was served as the third component of the parameter variable in Algorithm 3.2, where $\omega \in [\omega _0, \omega ^*]=[0.95, 3.30]$. In addition, we chose $N = 50, M=0.6, \widetilde{N} = 6000$, $\widetilde{\sigma }=0.1, k_0 = 0.01$ and $\Omega = (-6,6)^2$. The minimum eigenvalue of the LSE was detected at $\mu _1 \approx 1.9511$. Figure 7a depicts the ground state solution curve of the wave function and its projections on the three components using the two-norm with respect to the chemical potential $\mu$. Note that the two-norm of the component $\psi _{-1}$ was relatively small compared to that of the components $\psi _1$ and $\psi _0$ because of the affect of the magnetization *M*. Moreover, there was a turning point on the solution curve where the angular velocity $\omega = 0.95$, and the chemical potential $\mu = 28.0693$, Fig. 7b displays the contour plots of the three components where $\omega = 2.05, 2.81$, and 3.20. When $\omega = 2.05$, vortices of the component $\psi _1$ formed a hexagonal lattice, and the counterparts of the component $\psi _0$ formed a honeycomb, which was surrounded by a hexagonal lattice with yellow color. When $\omega = 2.81$, an inner ring of vortices was formed in the domain of $\psi _1$, which was surrounded by an outer ring of vortices. Yet a central vortex and an outer ring of vortices was observed in the domain of $\psi _0$. But the size of the ring of vortices was smaller than the counterpart of $\psi _1$. When $\omega = 3.04$, a central vortex was formed in the domain of $\psi _1$, and the outer ring of vortices remained there. The phenomenon observed in the domain of $\psi _0$ was similar to that in $\psi _1$ but with smaller size. In all cases no vortices was found in the domain of $\psi _{-1}$.

### Example 4.5

**Figure 8:**
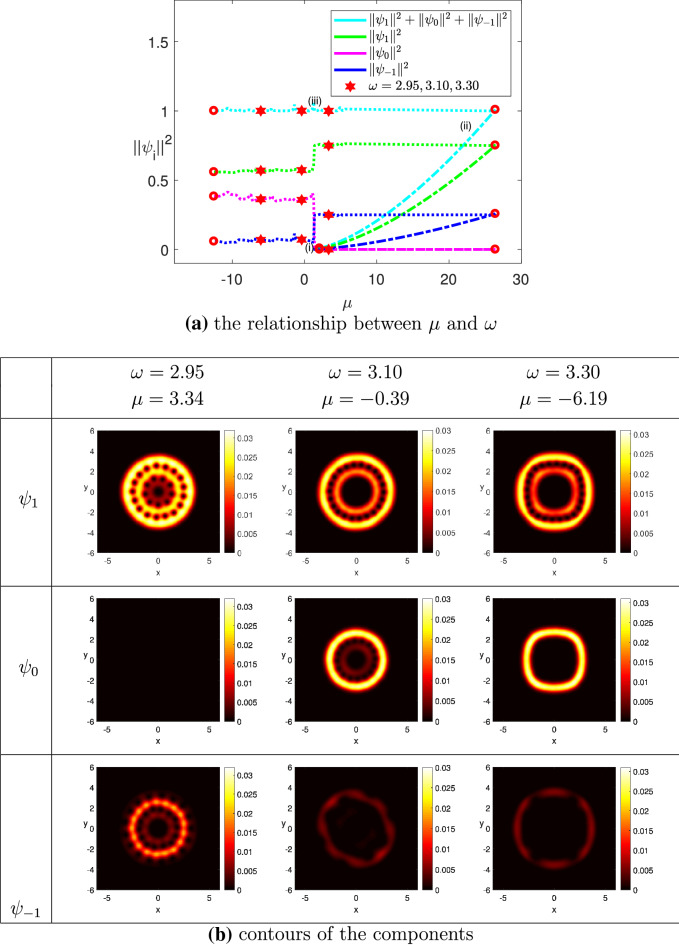
The ground state solution of $^{23}Na$, where $k_0 = 0.01$, $M=0.5$, $\widetilde{N} = 20{,}000$, and $\omega ^*=3.50$.

(The ground state solution of rapidly rotating spin-1 BEC for $^{{23}}$Na) We chose the same trapping potential as in Example 4.4, and used the angular velocity $\omega$ as the third component of the parameter variable, where $\omega \in [\omega _0, \omega ^*]=[0.95, 3.50]$. Moreover, we chose $N = 50$, $M=0.5$, $\widetilde{N} = 20{,}000$, $\widetilde{\sigma } = 0.1$, $k_0 = 0.01$ and $\Omega = (-6,6)^2$. The minimum eigenvalue of the LSE was detected at $\mu _1 \approx 1.9511$. Figure 8a displays the ground state solution curve of the wave function $\Psi$ and its projections on the three components by using the squares of the two-norm with respect to the chemical potential $\mu$. Moreover, a turning point was found on the solution curve where $\omega = 0.97$, and the chemical potential $\mu = 26.2572$. Figure 8b presents the contour plots of the three components, where $\omega =2.95$, 3.10, and 3.30. When the angular velocity $\omega =2.95$, two rings of vortices together with a central vortex were formed in the domain of the component $\psi _1$. Yet a central vortex surrounded by a ring with red color showed up in the contour of the component $\psi _{-1}$. Compared to the contours of $\psi _1$ and $\psi _{-1}$, the two-norm of the component $\psi _0$ is relatively small, namely, almost equal to zero. When $\omega = 3.10$, only the outer ring remained there with denser vortices in the domain of $\psi _1$, and the location of the inner ring of vortices and the central vortex was occupied by a bigger central vortex, which was surrounded by a ring with red color. But the two-norm of $\psi _{-1}$ became relatively small compared to that of $\psi _1$ because of the affect of the magnetization. However, we still could observe that a central vortex was surrounded by a ring with dark red color. On the other hand, a central vortex bigger than that of the component $\psi _1$ was formed in the domain of the component $\psi _0$, which was also surrounded by a ring with yellow color. When $\omega =3.30$, the contours of the three components were similar to those of the components when $\omega = 3.10$.

## Conclusions

We have applied the Lyapunov–Schmidt reduction to show the existence of nontrivial solution curves branching from eigenvalues of the linearized CGPEs. Based on the existence theory a multilevel pseudo-arclength continuation algorithm has been proposed which can efficiently compute the ground state solutions of rapidly rotating spin-1 BEC for both $^{87}Rb$ and $^{23}Na$. Our numerical results have demonstrated that various types of vortex lattices could be obtained for both $^{87}Rb$ and $^{23}Na$.

We remark the phenomenon exhibited in Example 4.2(ii). Owing to the repulsive interspecies interaction, it is intuitional that vacancies like vortices in one component are filled by droplets in another in order to lowering the energy of the system. However, the spinor degrees of freedom can provide a platform to study topological quantum phenomena in such multi-component system. This kind of BEC are called spinor BEC. For a spinor $F = 1$ BEC, the individual topological defects, half-quantum vortices in the polar phase, polar-core vortices, skyrmions (in 3D), and baby-skyrmions (in 2D) in the ferromagnetic phase have been discussed in^75,76^.

From Lemma 3.1 we may conclude that the magnetization *M* plays a key factor which makes Eq. (10) reduce to a single GPE when $M = \pm 1$. The contours displayed in Figs. 4, 7, and 8 verify numerically when $M \ge 0.5$, Eq. (10) almost decays to a two-coupled GPEs. As we increase the magnetization from $M = 0.5$ gradually to $M = 1$, it is expected that the two-coupled GPEs will decay to a single GPE. Finally, it would be of interest yet challenging to propose numerical methods for the ground state solution of the SPGPE for future studies.

**Table 2:** The chemical potentials, the values of *m* and *Order* of the convergence rate and convergence order, respectively, and the execution time for $^{87}Rb$ obtained by implementing Algorithm 3.2, where $\mu ^*=60.2127966$.

*N*	$\mu ^{(N)}$	$|\mu ^{(N)}-\mu ^*|$	*m*	*Order*	Time (s)
32	60.2230236	1.022703e−02	–	–	33.7165
64	60.2128598	6.328200e−05	0.158912	7.336377	46.7350
128	60.2127966	4.768800e−09	0.148332	13.695881	124.1998
256	60.2127966	0	$\infty$	$\infty$	418.4529
512	60.2127966	0	–	–	2682.4366
1024	60.2127966	0	–	–	13890.1136

**Table 3:** The chemical potentials, the values of *m* and *Order* of the convergence rate and convergence order, respectively, and the execution time for $^{23}Na$ obtained by implementing Algorithm 3.2, where $\mu ^*=25.3846144$.

*N*	$\mu ^{(N)}$	$|\mu ^{(N)}-\mu ^*|$	*m*	*Order*	Time (s)
32	25.3943102	9.6958505e−03	–	–	10.6860
64	25.3846771	6.2763477e−05	0.157502	7.271298	11.1034
128	25.3846144	2.3231195e−10	0.195419	18.043502	12.8372
256	25.3846144	0	$\infty$	$\infty$	15.6312
512	25.3846144	0	–	–	66.3975
1024	25.3846144	0	–	–	366.9496
